# mSphere of Influence: The ever-expanding universe of parasite cell biology

**DOI:** 10.1128/msphere.00043-25

**Published:** 2025-03-04

**Authors:** Benjamin Liffner

**Affiliations:** 1 School of Biological Sciences, Faculty of Science, Engineering, & Technology, University of Adelaide, Adelaide, South Australia, Australia; 2 Institute for Photonics and Advanced Sensing (IPAS), University of Adelaide, Adelaide, South Australia, Australia; Virginia-Maryland College of Veterinary Medicine, Blacksburg, Virginia, USA

**Keywords:** expansion microscopy, *Plasmodium*, apicomplexan parasites, electron microscopy

## Abstract

Ben Liffner studies the cell biology of apicomplexan parasites. In this mSphere of Influence article, he reflects on two key papers: “Three-dimensional ultrastructure of *Plasmodium falciparum* throughout cytokinesis” by R. M. Rudlaff, S. Kraemer, J. Marshman, J. D. Dvorin, et al. (PLoS Pathog 16:e1008587, 2020, https://doi.org/10.1371/journal.ppat.1008587) and “Expansion microscopy provides new insights into the cytoskeleton of malaria parasites including the conservation of a conoid” by E. Bertiaux, A. C. Balestra, L. Bournonville, V. Louvel, et al. (PLoS Biol 19:e3001020, 2021, https://doi.org/10.1371/journal.pbio.3001020). These two studies provided Ben with the conceptual framework to understand how parasites are organized in three dimensions, and the technique of ultrastructure expansion microscopy that he has since used to investigate this intriguing area of biology.

## COMMENTARY

Single-celled apicomplexan parasites have to grow, replicate, and divide all of the organelles of a eukaryotic cell all while being a tiny fraction of the size of most other eukaryotes. For example, a merozoite, the red blood cell invasive stage of the malaria parasite *Plasmodium falciparum*, contains a nucleus, Golgi, endoplasmic reticulum, mitochondrion, and a range of other organelles, but packages it all into a cell with a diameter only slightly longer than a single *Escherichia coli* bacterium. With all the complexity of eukaryotic cell packaged into a tiny volume, it has historically been very challenging to interrogate the cell biology of malaria parasites using conventional light microscopy. This article discusses two studies: “Three-dimensional ultrastructure of *Plasmodium falciparum* throughout cytokinesis” by Rudlaff et al. (1) and “Expansion microscopy provides new insight into the cytoskeleton of malaria parasites, including the preservation of a conoid” by Bertiaux and Balestra et al. (2), which utilized innovative microscopy approaches to enable new understanding of malaria parasite cell biology. These studies have had a profound influence on my science, both conceptually and technically, fundamentally changing the questions we can both ask and answer about parasite cell biology.

Both studies conceptually take a similar approach, recognizing the difficulty of imaging malaria parasites by conventional light microscopy. Instead, they ask: what can be learned about malaria parasite biology by applying advanced imaging modalities? Rudlaff et al. employ focused ion beam scanning electron microscopy (FIB-SEM) that involves serially milling and imaging of a sample to generate a high-resolution (~10 nm, *xyz*) three-dimensional reconstruction (1, 3). Utilizing FIB-SEM, Rudlaff et al. imaged asexual blood-stage malaria parasites as they underwent cell division (termed segmentation) (1). In doing so, the authors uncovered key insights into the processes of nuclear fission (karyokinesis), organelle biogenesis (rhoptries), and organelle fission (mitochondria and apicoplast) among others (1). More importantly, though in my opinion, the authors captured high-resolution holistic snapshots of parasites that reveal an unbiased view of a whole range of parasite organelles and structures in a near-native context. Bertiaux and Balestra et al. applied a technique called ultrastructure-expansion microscopy (U-ExM) to malaria parasites for the first time (2). U-ExM results in the physical expansion of the parasites ~4.5-fold while preserving parasite ultrastructure (4), effectively providing the equivalent of a 4.5-fold increase in image resolution. After developing U-ExM for malaria parasites, Bertiaux and Balestra et al. utilized the technique to study the parasite cytoskeleton in a number of different lifecycle stages. Most notably, the authors identified an “apical tubulin ring” in ookinetes, a motile lifecycle stage inside the mosquito midgut. The authors went on to show that this structure represented a conoid, a structure present in other apicomplexan parasites but previously thought to be absent in malaria parasites. During their characterization of the ookinete conoid, the authors made use of N-hydroxysuccinimide ester (NHS ester) fluorescent dyes, which promiscuously bind proteins and enable the visualization of a range of parasite structures without the need for protein-specific antibodies. Subsequently, NHS ester has been adopted widely to visualize a range of structures within malaria parasites for which antibodies do not exist or are not commercially available (5–7).

Both studies were published in mid-2020 (as a preprint [2]) in the final few months of my PhD, a time when I was contemplating what was next for me. As much of my research was based on microscopy, I had spent a lot of time thinking about how malaria parasites looked, but until reading the study by Rudlaff et al. (1), it was as if my vision had been blurred. All of a sudden, I had a clear mental picture of how malaria parasites were organized in three dimensions, and alongside that, a swathe of new biological questions I wanted to answer. But FIB-SEM is technically challenging, low throughput, and a technique I had no experience with. Expansion microscopy has been around in various forms since 2015 (8), but it was a technique I was unaware of until ultrastructure-expansion microscopy was first applied to *Toxoplasma gondii* (9). From this, I realized that U-ExM might be a useful technique for me but due to my lack of familiarity with *Toxoplasma* at the time, I failed to recognize the power of the technique. From the first figure in the Bertiaux and Balestra et al. study (2), however, it was clear to me that U-ExM was a game changer. Previously non-descript fluorescent dots reveal themselves as highly intricate biological structures. By combining the conceptual insights provided by Rudlaff et al. with the U-ExM applied by Bertiaux and Balestra et al. (2), I now had the biological questions I wanted to tackle and the technique to make it happen.

While FIB-SEM has been an extraordinarily useful tool in understanding malaria parasite cell biology, only a small number of studies have utilized this technique since the publication of Rudlaff et al. (1) in 2020. These studies have investigated lipid droplets and organelle fission in asexual blood-stage parasites (10, 11), nuclear and cell division in oocysts (12), and developed a comprehensive ultrastructural map of gametocytes (13). By contrast, U-ExM has found widespread use among the malaria community being used in at least 40 studies since the publication of the Bertiaux et al. paper. These studies have used U-ExM on *P. falciparum*, *Plasmodium berghei* (14), and *Plasmodium yoelii* (15) and covered nearly every lifecycle stage, including imaging oocysts and sporozoites in their mosquito host *in situ* (16). This application of U-ExM to malaria parasites has been used to investigate biological processes as broad as host cell invasion (6), organelle fission (7), endocytosis (17), mitosis (18), organelle biogenesis (7), and cell division (19), highlighting the utility of this technique for interrogating all aspects of parasite cell biology. With the application of FIB-SEM to different parasite lifecycle stages and ongoing development of expansion microscopy protocols, it is clear that these techniques are here to stay and will continue to shape our understanding of the unique and intricate cell biology of malaria parasites.
